# A strong-field driver in the single-cycle regime based on self-compression in a kagome fibre

**DOI:** 10.1038/ncomms7117

**Published:** 2015-01-27

**Authors:** T. Balciunas, C. Fourcade-Dutin, G. Fan, T. Witting, A. A. Voronin, A. M. Zheltikov, F. Gerome, G. G. Paulus, A. Baltuska, F. Benabid

**Affiliations:** 1 Institute of Photonics, Vienna University of Technology, Gusshausstrasse 27/387, 1040 Vienna, Austria; 2 GPPMM Group, XLIM Research Institute, CNRS UMR 7252, University of Limoges, 87060 Limoges, France; 3 Blackett Laboratory, Imperial College, London SW7 2AZ, UK; 4 Department of Physics, International Laser Center, M.V. Lomonosov Moscow State University, Vorob'evy Gory, Moscow 119992, Russia; 5 Russian Quantum Center, Skolkovo, Skolkovo, Moscow Reg., 143025, Russia; 6 Department of Physics and Astronomy, Texas A&M University, College Station, TX 77843, USA; 7 Kurchatov Institute National Research Center, ploshchad' Akademika Kurchatova 1, 123098 Moscow, Russia; 8 Institute of Optics and Quantum Electronics, Friedrich-Schiller-University, Max-Wien-Platz 1, D-07743, Jena, Germany

## Abstract

Over the past decade intense laser fields with a single-cycle duration and even shorter, subcycle multicolour field transients have been generated and applied to drive attosecond phenomena in strong-field physics. Because of their extensive bandwidth, single-cycle fields cannot be emitted or amplified by laser sources directly and, as a rule, are produced by external pulse compression—a combination of nonlinear optical spectral broadening followed up by dispersion compensation. Here we demonstrate a simple robust driver for high-field applications based on this Kagome fibre approach that ensures pulse self-compression down to the ultimate single-cycle limit and provides phase-controlled pulses with up to a 100 μJ energy level, depending on the filling gas, pressure and the waveguide length.

## Abstract (web summary)

Single-cycle and sub-cycle field transients are typically generated by external pulse compression where a combination of nonlinear broadening followed up by dispersion compensation is used. Here, Balciunas *et al*. use self-compression in a Kagome fibre to generate phase-controlled single-cycle pulses.

## Graphical abstract

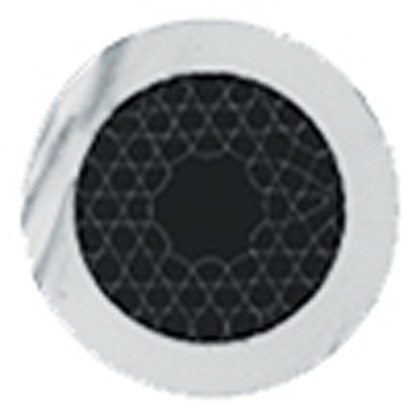

Spectral continua supporting single-cycle operation are routinely obtained in nonlinear waveguides and in self-guided free-space femtosecond filaments. These can be dimensioned to accommodate high energies, with hollow capillaries^1^,^2^,^3^ and filaments^4^ capable of handling millijoule pulses. However, because of the challenges of nonlinear interaction in the high-energy regime, pulse recompression after broadening is either handled in a separate set-up^5^ or, as in the case of filamentation, may lead to a self-compression of a small energy fraction^6^,^7^,^8^,^9^,^10^. Self-compression in a large-core Kagome-lattice microstructured fibre^11^,^12^ has been recently suggested as a solution for handling microjoule energy pulses.

The ability of ultrashort laser pulses to self-compress in negative dispersion waveguides is widely known since the observation of the optical soliton over 30 years ago^13^. Solid-core photonic crystal fibres are particularly well suited for accurate dispersion shaping and controlled solitonic self-compression^14^,^15^; however, they are not scalable for high-energy operation as the correct dispersion profile generally corresponds to tiny core diameters. Second, the material damage threshold sets a severe limit on the maximum pulse energy^16^. Gas-filled capillaries permit high-energy throughput and are routinely used for microjoule-level few-cycle pulse generation^1^; however, the negative dispersion is weak and is usually overtaken by the positive dispersion of the gas thus preventing self-compression. Finally, the advent of photonic bandgap (PBG)-guiding hollow-core photonic crystal fibre (HC-PCF)^17^ showed promise to overcome these limitations of hollow capillaries following the pioneering demonstration of megawatt peak power solitons in this type of fibres^18^,^19^. However, restrictions originating in the PBG-guiding mechanism and intrinsic problems of the HC-PCF performance have imposed difficulties in terms of a limited transmission bandwidth, high dispersion slope and multimode structure^20^, thus hampering the quest for nonlinear compression of high-energy broadband pulses. The advent of inhibited coupling (IC) guiding HC-PCF created a new paradigm in optical guidance, whereby core-guided modes could cohabitate with cladding modes with no significant interaction between the two types of modes^21^. Unlike the earlier PBG-guided HC-PCF, the ones with IC^22^ stand out with a significantly larger bandwidth, lower dispersion and higher power handling, opening the opportunity to reach very high intensities capable of ionizing the gas in the fibre core^23^,^24^. Furthermore, and since the seminal introduction of hypocycloid core-contour (that is, with negative curvature) in the fibre design^16^, IC guiding HC-PCF exhibits now comparable transmission loss as PBG HC-PCF, with record loss of 17 dB km^−1^ ~1 μm (refs 25, 26).

Here we report on the scalable self-compression scheme that relies on a specialty photonic crystal fibre with a large hollow core filled with a noble gas as a nonlinear optical medium. The potential of the generated waveform is verified by a typical high-field experiment^27^ on photoelectron emission in above-threshold ionization (ATI)^27^. The compression dynamics is based on a modified solitonic regime in which an optical shock wave enhances spectral broadening and the energy buildup in the temporally compressed part of the pulse proceeds to an unconventionally high level. The enhanced solitonic compression scenario is enabled by a fortunate combination of the wave-guiding mechanism, optical nonlinearities and dispersion properties of a hypocycloid core Kagome-lattice HC-PCF^16^. We show both experimentally and theoretically, that, in a single step, ultrashort infrared pulses with energies of several tens of microjoules undergo a 20-fold nonlinear self-compression to reach the pulse duration of 4.5 fs full-width at half-maximum, below the optical period of 5 fs and a gigawatt peak power at the fibre exit. We applied rigorous pulse characterization techniques based on stereo-ATI and SPIDER (spectral phase interferometry for direct electric field reconstruction^28^, Supplementary Methods section ‘Pulse generation and characterization experimental set-up’ and Supplementary Figs 1,2) to attest that a subcycle duration of the self-compressed pulse indeed can be reached. This Kagome-fibre-driven and single-step self-compression scheme allow a great simplification of the attosecond and field-sensitive measurements, such as ATI electron spectrometry, coincidence momentum imaging and THz generation in plasma.

## Results

### Pulse self-compression scheme

**Figure 1:**
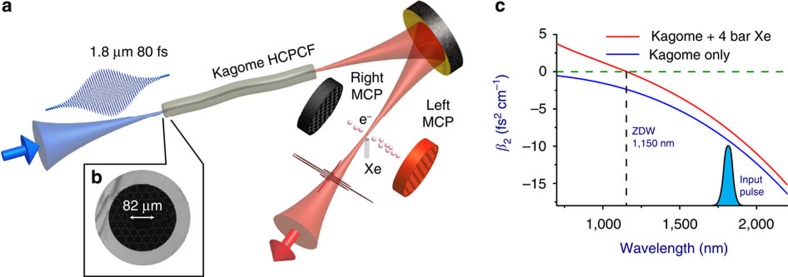
Pulse self-compression scheme based on inhibited coupling guiding HC-PCF and the field measurement using stereo-ATI photoelectron spectrometry. (**a**) The experiment consists of launching infrared 80-fs pulses with energies of up to 120 μJ into 0.2-m-long hypocycloid-core Kagome HC-PCF with an inner circle of 82 μm (corresponding to a mode-field diameter of ~64 μm). The pulses were generated in an optical parametric amplifier, and which wavelengths were tuneable in the 1.4- to 1.9-μm range. The fibre shown in the inset (**b**) was optimized for this spectral range, exhibiting a linear loss^31^ of ~70 dB km^−1^ and an S-shaped dispersion curve over 1,000–2,000 nm optical bandwidth and featuring anomalous dispersion ranging from 0 to 0.5 ps km^−1 ^nm^−1^ within the spectral range of 1,150–2,000 nm as shown in Supplementary Fig. 11. The solitonic self-compression in the negative dispersion regime is achieved by filling the fibre with Xenon to a pressure of 4 bar so as to provide the necessary optical nonlinearity. At such a low pressure, the dispersion is dominated by the negative dispersion of the waveguide down to zero dispersion wavelength (ZDW) of 1,150 nm as shown in **c**. The pulses were then refocused into stereo-ATI electron spectrometer with two microchannel plate (MCP) detectors.

Here we show that the optical shocks of subcycle duration^29^,^30^ can be generated experimentally in a single guided-photonic structure. Shockwaves are widespread in the Nature and generally lead to generation of extremely sharp temporal features. We exploit the latest advances in the IC guiding HC-PCF design that have led to a lower transmission loss and a smaller power fraction propagating inside the glass walls^16^ much improved single-mode operation^31^, and a controllable negative dispersion (see Fig. 1c and Supplementary Fig. 11). We identify and experimentally demonstrate that a regime of optical pulse compression, in which an optical shock wave, arising as a part of the highly nonlinear-guided-wave evolution of an ultrashort laser pulse, enhances solitonic pulse compression. Subcycle field waveforms, generated as a result of this dynamics, combine the most striking features of optical shock waves on one hand and solitons on the other. Most crucially from the viewpoint of practical high-intensity applications, this unique interplay of solitonic and shock-wave phenomena was achieved at intensity level above 10^14 ^W cm^−2^ and the peak power level above 1 GW, the combination of which have not been demonstrated simultaneously for an optical fibre. Remarkably, this dynamics takes place in the waveguide geometry, in the single-cycle self-compression limit and in a single spatial mode. A practical advantage is the energy and spectral scalability of this system determined by the ionization potential, *I*_p_, of the gas filling the waveguide and the laser wavelength as the core diameter increases linearly with wavelength and the transmitted energy increases quadratically. Finally, by refocusing the self-compressed 10^14 ^W cm^−2^ beam from the fibre, the scheme can serve as a source for strong-field applications. To emphasize that such a single-cycle pulse source is capable of handling real-world applications, we employ ATI of Xe atoms in a stereo time-of-flight spectrometer^32^,^33^. This model experiment is useful in many respects. First, a successful ATI measurement unequivocally proves that the source operates in the strong-field regime. Second, it permits a full characterization of the electric field of the laser pulse in every laser shot, and the large asymmetry of the electron spectrum signify a single dominant half cycle within the pulse. Third, it allows verification of the carrier envelope phase stability of the solitonically self-compressed pulses.

### Experimental set-up

The simple Kagome-fibre-based pulse post-compression scheme is illustrated in Fig. 1. Input 80-fs pulses centred at 1.8 μm are launched into a negative dispersion waveguide where they undergo self-compression. The Kagome fibre is placed inside a high-pressure gas cell capped on the output side with a quartz window that is only 0.2 mm thick to minimize distortions of the out-coupled self-compressed pulse. Note that under our experimental conditions some small amount of residual positive chirp (≈18 fs^2^) is acquired during self-compression, which is compensated by the negative dispersion of the gas cell window (see Supplementary Fig. 6). To eliminate additional dispersive broadening, only reflective optics is used downstream for conventional and ATI-based pulse characterization.

### Characterization of self-compressed pulses

**Figure 2:**
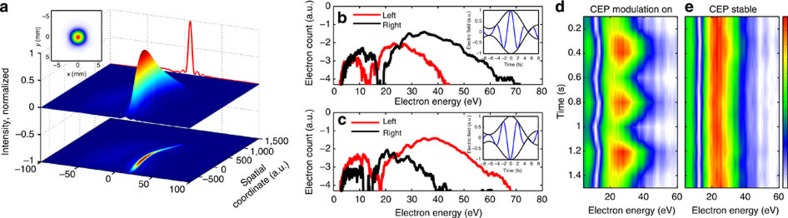
Characterization of the self-compressed pulse using various techniques. The spatial uniformity and whole-beam compression are illustrated in the spatiotemporal reconstruction of the pulse depicted in (**a**) and measured beam profile shown in the inset. The panels (**b**,**c**) show the photoelectron spectrum of ionized Xe atoms in two directions in stereo-ATI spectrometer using self-compressed pulses for +cos and −cos electric fields. The panels (**d**,**e**) show that the self-compressed pulses retain the CEP phase of the much longer input pulses despite the very high compression ratio. The CEP of the input pulses was locked and the dependence of the electron spectrum was measured when the CEP of the laser is (**d**) modulated with a liner phase ramp scanned with the speed of 12 rad s^−1^ and (**e**) kept stabilized.

**Figure 3:**
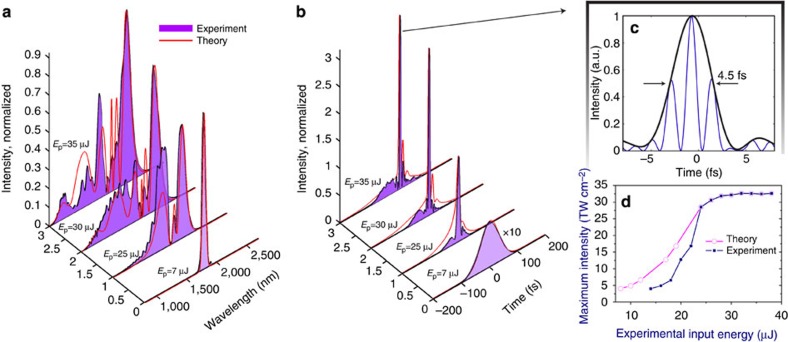
Measured pulse self-compression for different input energies. (**a**) Experimentally measured spectra after the waveguide. (**b**) Output pulse profiles measured with SEA-SPIDER technique (**c**) Intensity (red) and instantaneous field intensity (blue) profiles of the shortest self-compressed pulse. (**d**) Measured and calculated dependence of the self-compressed pulse peak intensity at the output window. Measurements were performed for 0.2 m fibre filled with 4-bar Xenon.

Complete temporal, spectral and spatial characterization of the input and output laser waveforms was performed using the technique of spatially encoded SEA-SPIDER. The key results of the SEA-SPIDER and stereo-ATI measurements of the self-compressed pulses are presented in Fig. 2. The experimentally obtained spatiotemporal pulse intensity distribution shown in Fig. 2a proves that the self-compression mechanism is indeed active across the entire beam cross-section and is not limited to its central portion—one of the key strengths of the demonstrated method for nonlinear pulse compression of high-energy pulses. Figure 2b,c shows ATI spectra obtained for different Carrier Envelope Phase (CEP) settings (with a phase difference of *π*) of the 60 μJ output pulses obtained when the cell is filled with 1 bar of Ar. The dramatic asymmetry in the flux of the photoionized electron outgoing in the direction of the highest-intensity half-cycle^34^ of the laser pulse serves as a direct proof that the electric field of the pulse carries but a single dominant half-cycle. Using the procedure described in the Supplementary Methods (section ‘Characterization of the self-compressed pulses with stereo-ATI electron spectrometry’) and Supplementary Fig. 8, the measured stereo-ATI spectra allow us to calibrate the CEP values and the actual peak intensity, 5 × 10^13 ^W cm^−2^, that was reached in the ATI apparatus. By engaging an active CEP lock on the laser pumping the IR OPA that supplies 1.8 μm input pulses, we were also able to verify that CEP stability is preserved in the self-compressed output pulses as confirmed by an ATI measurement shown in Fig. 3d,e (see Supplementary Methods for details).

An experimentally measured as well as a numerically modelled evolution of output pulse temporal and spectral profiles after the propagation in the HC-PCF is shown in Fig. 3 as a function of input pulse energy. The dynamics reveal pulse self-compression in a negative dispersion fibre down to subcycle duration. FROG and SEA-SPIDER techniques were used to characterize the initial stages of the self-compression down to few-cycle regime to independently confirm the same result. However, with the spectra ultimately spreading into the multioctave range, only the SEA-SPIDER technique remained adequate pulse characterization in the single-cycle regime.

As a consequence of the over-an-octave bandwidth, the red and blue wings of the spectrum exhibit significantly different divergence, with longer-wavelength components diverging faster than the blue ones. For the pulse components near the beam axis, the faster loss of the spectral content in the red wing amounts to an effective pulse broadening in time. To limit such spectral filtering caused by different beam divergences, we used a spherical mirror to re-image the output of the fibre on the sum-frequency crystal and on the ATI gas target as sketched in Fig. 1a. Re-imaging of the fibre output mode on the target effectively reconstitutes all the spectral components and allows formation of a subcycle pulse that otherwise would have been purged to a full cycle pulse known to be the limit for a free-propagating pulse as a consequence of the Gauss theorem.

Because the ionization regime can be reached by the self-compressed pulse inside the Kagome waveguide itself, the energy of the single-cycle pulse critically depends on the choice of noble gas. The highest output pulse energy, 75 μJ, was reached for Ar because of its high ionization potential. These pulses were capable of driving the ATI apparatus (Fig. 2b,c), although their duration was not as short as in the case of other gases. The highest quality self-compression results, summarized in Fig. 3, were achieved for the gas with the highest nonlinearity, Xe. As a consequence of its lower ionization potential, we had to restrict the maximum usable input energy to 35 μJ resulting in the highest output energy of 25 μJ corresponding to a subcycle pulse.

These subcycle pulses result from an original sequence. As demonstrated in Fig. 3, at low input pulse energies, the pulse starts to shorten because of the interplay between the negative group-velocity dispersion (GVD) and the self-phase modulation. At progressively higher input pulse energies, the nonlinear spectral broadening is increased and the resultant pulse duration is reduced approximately linearly with the pulse energy, as depicted in Fig. 3d). Starting with energies ~26 μJ, a needle-like structure emerges on the pulse. Subsequent dynamics differs significantly from the conventional high-order solitonic pattern because the pulse continues to shorten instead of breaking up. Note that the measurement of the pulse evolution and the monotonic pulse shortening show that the spectrum is coherent and single ultrashort pulse is formed, and the coherent artifacts due to measurement technique^35^ can be neglected. Numerical modelling convincingly shows that the breakup is constrained because of the higher-order nonlinear terms. At 35 μJ input pulse energy, a 4.5-fs pulse is formed. Further increase in the energy does finally break the pulse up. The pedestal of the pulse is intrinsic to the self-compression process. The main pulse contains around 50% of the energy at the shortest 4.5-fs pulse case. At the expense of slightly sacrificing the pulse compression ratio, the pulse fidelity can be substantially improved such that ~60–70% of the energy is gathered in the main pulse. The fibre length of 0.2 m was chosen to achieve the required nonlinear length at a safe operational gas pressure in the fibre cell (see Supplementary Methods section ‘Optimization of self-compression and energy scalability’ for details). Higher soliton numbers enable compression to shorter pulse widths within shorter stretches of fibre. On the other hand, the input pulse energy and peak intensity are limited by the ionization of the noble gas. As expected, Xe provided the best self-compression quality because of the highest nonlinearity versus ionization rate ratio and—as a consequence of being an atomic gas—absence of a delayed Raman response.

## Discussion

The intensity in the fibre is already in the strong-field regime, that is, the self-compressing pulse begins to ionize the gas. Consequently, the use of a gas (Xe) with a low-ionization potential limited the maximum usable energy of the self-compressed pulse in this waveguide to ~40 μJ. At higher energies, plasma dispersion effects ruin the self-compression mechanism. Despite the low energy, the pulses compress to a single cycle and at a GW peak power level. For the cosine electric field pulse where the peak of the envelope coincides with the peak of the electric field, the instantaneous intensity of the adjacent electric field peaks is 50% less than the main peak. For the extreme nonlinear processes like strong-field ionization or high-order harmonic generation, this confines the interaction to the single main peak of 1.4-fs duration.

**Figure 4:**
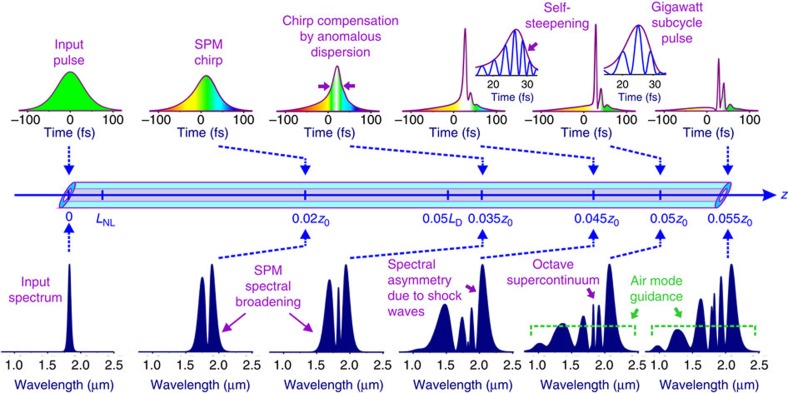
Different phases of the pulse self-compression in a waveguide. On the propagation scale of the nonlinear length *L*_NL_, self-phase modulation (SPM) induces a chirp and gives rise to noticeable spectral broadening of the laser field.

The experimental measurement results are in good agreement with theoretical simulations that allow getting more insight into the physics behind the self-compression process. Figure 4 outlines the stages of soliton pulse self-compression down to a subcycle pulse width. More in-depth measurement data and theory calculations are provided in Supplementary Figs 3,4. In the case of octave-wide bandwidths, the nonlinear dynamics of the pulse shape deviates substantially from the classical soliton dynamics picture. In the regime explored in our work, the dispersion length *L*_D_ in this regime is ~290 times larger than the nonlinear length *L*_NL_=(*γ*_0_*P*_0_)^−1^ (here *γ*_0_=2*πn*_2_/(*λ*_0_*S*_eff_) is the nonlinearity parameter, *n*_2_ is nonlinear refractive index, *λ*_0_=2*πc*/*ω*_0_ is the central wavelength of the input pulse, *S*_eff_ is the effective fibre mode area and *P*_0_ is the peak power of the input pulse) corresponding to a soliton order *N*=(*L*_D_/*L*_NL_)^1/2^≈17 (here 
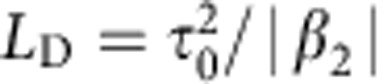
, *τ*_0_ is the input pulse width and *β*_2_ is the GVD coefficient, dispersion profile polynomial is provided in Supplementary Notes). With such a high nonlinearity and mild dispersion, self-phase modulation has enough time and space to generate a large nonlinear phase shift and dramatically broaden the spectrum before dispersion effects start to play any noticeable role in the solitonic dynamics. At around *z*≈0.05*z*_0_, ≈0.08L_D_ (*z*_0_=*πL*_D_/2 is the soliton period) dispersion starts to compress the central part of the pulse, where the chirp is almost linear. As the pulse width becomes close to a single field cycle at *z*≈0.045*z*_0_, shock-wave effects come into play (see Supplementary Fig. 5), blue-shifting the spectra and steepening the trailing edge of the field waveform. Following the point of maximum compression, achieved at *z*≈0.05*z*_0_, self-steepening generates an intense blue wing of the spectrum, which eventually falls beyond the zero GVD wavelength. Pulse compression in this regime is thus enhanced by self-steepening and is limited by the finite range of air-mode guidance in the gas-filled fibre core.

In summary, we demonstrate an efficient pulse self-compression scheme based on shock-wave formation in an ultrabroadband Kagome-lattice fibre. The proposed approach leads to the subcycle pulse generation in a relatively simple experimental set-up. The scheme is scalable in energy and wavelength and has the potential to answer the demand for energetic subcycle sources in many strong-field applications.

## Methods

### Configurations of the strong-field source

**Figure 5:**
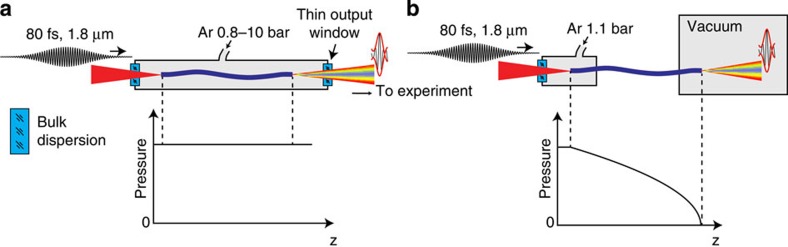
Schemes of pulse delivery to an application and corresponding pressure profiles along Kagome fibre. (**a**) Fibre mounted in a static gas cell and (**b**) a pressure gradient case when the output of the fibre is mounted directly in a vacuum chamber.

The source described in this paper is compatible with several geometries used in generic strong-field experiments. Specifically, one challenge is the delivery of the self-compressed pulse on the target and the ability of the nonlinear self-compression scheme to compensate extra dispersion between the fibre exit and the target to which the pulse has to be delivered at the highest possible level of compression. Here we assume that a dispersion-less focusing is employed by use of a concave mirror and distinguish two generic geometries suitable for a strong-field experiment, as summarized in Fig. 5. The first relevant geometry, shown in Fig. 5a, is based on a Kagome waveguide in a cell with a static gas pressure. At the minimum, this includes an inevitable extra dispersion of the out-coupling window. In some schemes, on top of that one might be also required to compensate additional dispersion contributions arising from propagation through air and an in-coupling window. The second scheme, shown in Fig. 5b, assumes fibre termination directly into a vacuum target chamber. The main conceptual difference between the two schemes is that the one with vacuum termination avoids extra bulk dispersion and supplies a longitudinal pressure gradient.

The case of extra bulk dispersion (Fig. 5a) is frequently encountered in gas-based self-compression schemes^36^,^37^ and can be turned into an advantage because the intensity of the divergent beam in the exit window is low and it can be treated as a linear bulk compressor. As pointed out in Supplementary Discussion, the bulk dispersion of the window in the case of our infrared wavelength is anomalous, that is, of the same sign as the gas-filled waveguide dispersion. Because of that, one can terminate the process of nonlinear self-compression at a safe peak power level and then additionally post-compress the pulse with bulk dispersion to reach a higher peak power level while avoiding additional nonlinear interaction. In the Supplementary Methods (section ‘Optimization of self-compression and energy scalability’) we explain how the interplay of the intensity, gas nonlinearity, gas pressure and the waveguide length can be used to optimize the compression level and the peak power. In a practical experiment, in which the gas type and the fibre length are fixed, we carefully tune the gas pressure and attenuate the input pulse energy to achieve the desired balance between the pulse energy and pulse fidelity and duration (see Supplementary Fig. 7). In the context of the experiments with Xe and Kr filling gases, we point out that the presence of small extra bulk dispersion allows us to increase the pulse energy on target compared with the case when the highest peak power would be reached inside the fibre, entailing ionization loss and associated pulse and beam distortions (see Supplementary Fig. 9). We conservatively estimate that the use of a 150 μm output window helps us win additional 10–15% of the usable pulse energy and an even larger fraction of the achievable peak power (*cf*. Supplementary Discussion) compared with the case when the self-compressed pulse exits the waveguide without a small amount of pre-chirp compensated by the window (see Supplementary Fig. 6).

The case of vacuum termination (Fig. 5b) closely resembles the pressure-gradient technique used in the hollow waveguide chirping technique for high-energy pulses^38^. The main advantage of this scheme is that the energy transmission can be significantly boosted in comparison with the static pressure case because the gas density is progressively reduced with the growth of the peak power, helping to hold off ionization losses.

Whereas the experiments presented in this paper were performed using the fibre in a static gas cell, we have also investigated the suitability of the scheme in the geometry shown in Fig. 5b by measuring the self-compressed pulse output spectrum shown in Supplementary Fig. 10.

## Author contributions

T.B., C.F.-D., G.F. and T.W. performed the experiments and analysed the data, F.B. designed the Kagome fibre, C.F.-D., F.B. and F.G. manufactured the fibre, A.A.V. and A.M.Z. developed the theoretical model and performed numerical simulations, G.G.P. contributed the apparatus, methodology and data analysis for the ATI photoelectron measurements, T.B., A.B., F.B. and A.M.Z. wrote the paper. F.B., A.Z. and A.B. initiated the project. All the co-authors discussed the results and contributed to the manuscript.

## Additional information

**How to cite this article:** Balciunas, T. *et al*. A strong-field driver in the single-cycle regime based on self-compression in a Kagome fibre. *Nat. Commun.* 6:6117 doi: 10.1038/ncomms7117 (2015).

## Supplementary Material

Supplementary Information Supplementary Figures 1-11, Supplementary Notes, Supplementary Discussion, Supplementary Methods and Supplementary References.
