# Supplementary material for: A strong-field driver in the single-cycle regime based on self-compression in a kagome fibre
Source: Nat Commun. 2015 Jan 27;6:6117. doi: 10.1038/ncomms7117 (PMC4317490; doi:10.1038/ncomms7117)
Supplement: Supplementary Information — Supplementary Figures 1-11, Supplementary Notes, Supplementary Discussion, Supplementary Methods and Supplementary References. [file ncomms7117-s1.pdf]

Supplementary material

Supplementary Figures

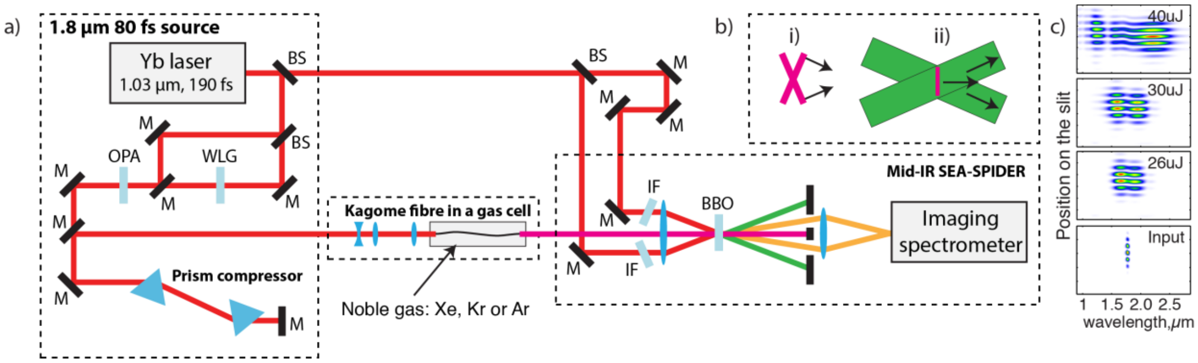

Supplementary Figure 1. a) A general scheme of the experimental setup and the mid-IR SEA-SPIDER pulse characterization apparatus. BS – beam splitter, M – mirror, IF – narrowband interference filter, WLG – white light generation in bulk YAG plate. b) an illustration of geometrical smearing effect in non-collinear SHG FROG measurements: i) two non-collinear beam crossing in the case of SHG FROG and ii) two long-pulse ancilla beams and a short test pulse fully overlap in the case SEA-SPIDER. c) raw SEA-SPIDER traces for several input pulse energies, pulses were self-compressed in 20 cm long fibre filled with 3 bar Xenon.

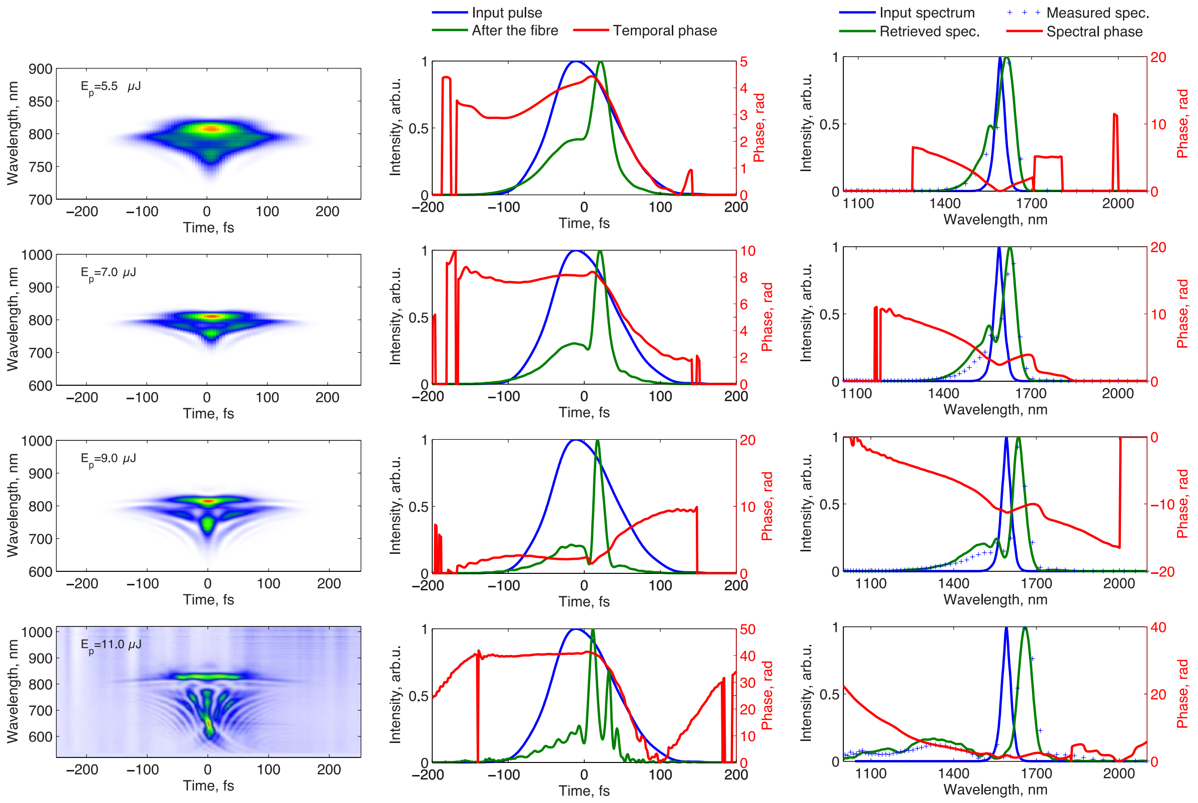

Supplementary Figure 2. SHG FROG traces, reconstructed temporal profiles and spectra of the self-compressed pulses for several input pulse energies. Input pulse parameters: $\lambda_0 = 1.6$ µm, 75 cm long fibre filled with 3 bar Krypton.

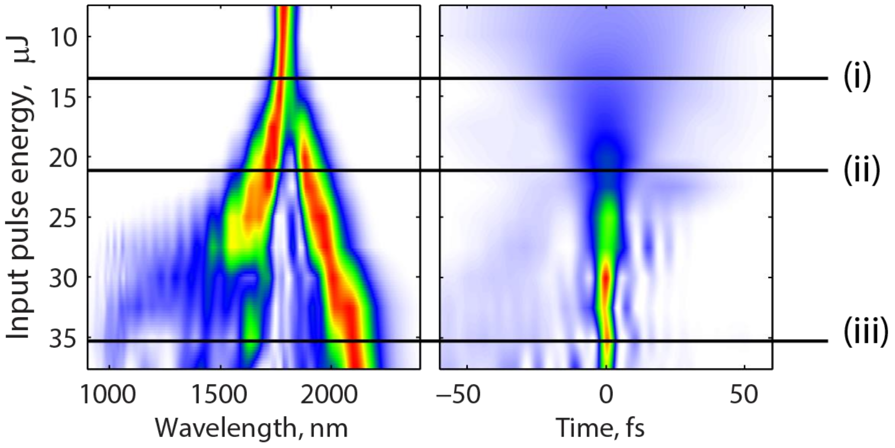

Supplementary Figure 3. Dependence of the self-compressed pulse spectrum and temporal profile on input pulse energy. Left panel shows the spectrum and the right panel shows the corresponding temporal pulse profile as measured using SEA-SPIDER technique. The dynamics can be divided into three phases: (i) classical high order soliton formation, where the SPM-induced nonlinear phase is balanced by the phase acquired due to the anomalous dispersion of the fibre and the input pulse power is several times the power of the fundamental soliton (ii) the spectrum of the self-compressed pulse increases enough so that the group velocity dependence on intensity steepens the trailing edge of the pulse, giving rise to an optical shock wave. (iii) an intense sub-cycle duration light transient is formed as a result of shock-wave-enhanced soliton self-compression. Measurements were done for 0.2 meter fibre filled with 4 bar Xenon.

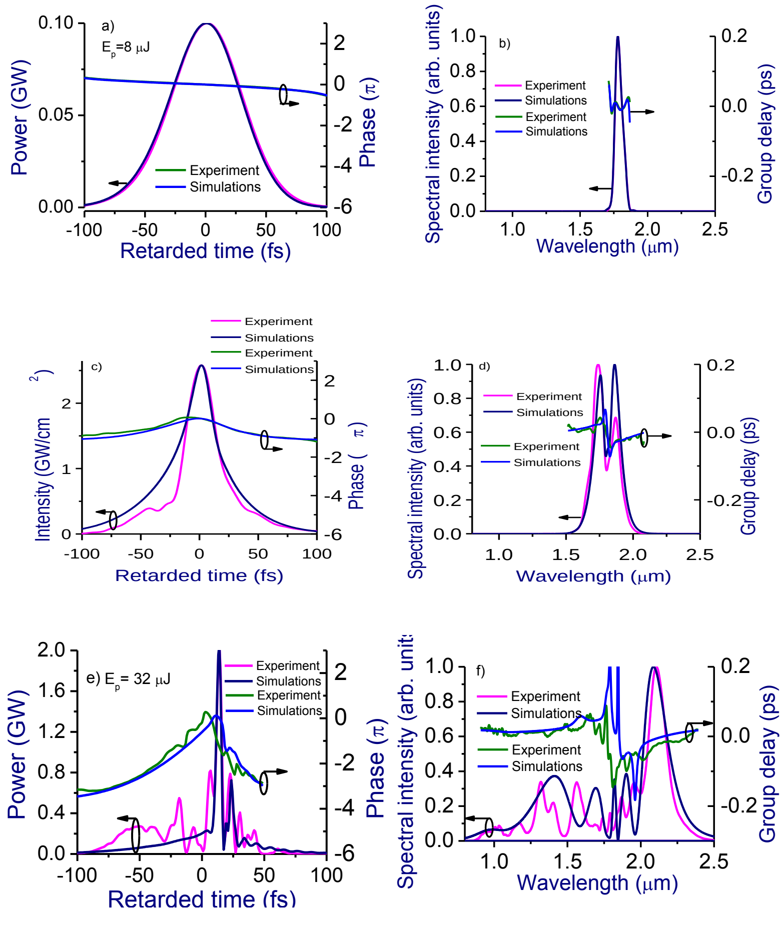

Supplementary Figure 4. A comparison of the evolution of the pulse spectrum and temporal profile during the self-compression process in the fibre to the calculations. Firstly, a broad spectrum is formed with a sharp excursion of the group delay at around the centre of the pulse spectrum as shown in panels c) and d). This sharp oscillation of the

group delay is flanked by the spectral wings in which the group delay changes relatively slowly. However, the blue- and the red-shifted spectral wings are distinctly separated in time. This delay between the blue and the red wings increases with the increase of the input pulse energy. However, further increase of the pulse energy reduces the lag between the blue and the red branches of the group delay. As a result, the blue and the red spectral shoulders are synchronized in time and an ultrashort temporal structure is formed, as shown in e) and f). These features are very reliably verified by the numerical modelling that reproduces an abrupt group delay excursion and thereby confirms the origin of the longer pedestal under the ultrashort self-compressed pulse transient which is associated with this group delay oscillation in the vicinity of the carrier frequency. Measurements and calculations were done for 0.2 meter fibre filled with 4 bar Xenon.

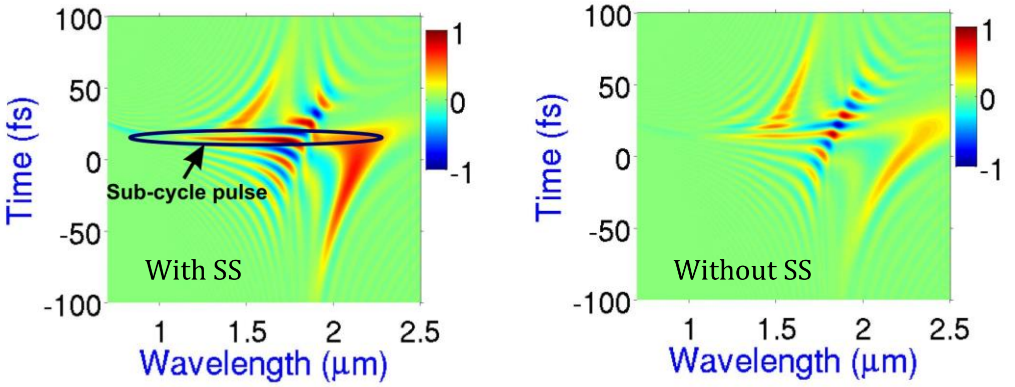

Supplementary Figure 5. Time-frequency analysis illustration of the effect of the shock-wave and self-steepening on sub-cycle pulse generation. The graph on the left shows the Wigner distribution of self-compressed pulses calculated with the full model and on the right with the self-steepening term switched off. Calculations were done for 0.2 meter fibre filled with 4 bar Xenon at the input pulse energy of 28 μJ.

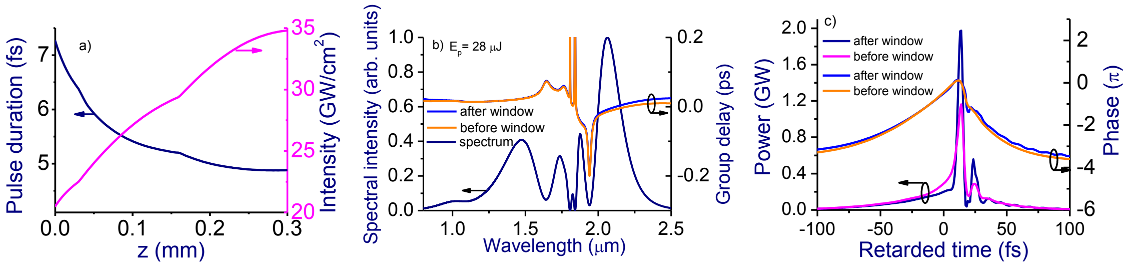

Supplementary Figure 6. Compensation of the residual chirp of the self-compressed pulse in a window. a) calculated pulse duration and peak intensity evolution during linear propagation through fused silica window. b) Spectral intensity and spectral phase before and after propagation through the window. c) pulse temporal profile and temporal phase before and after propagation through the window. Calculations were done for 0.2 meter fibre filled with 4 bar Xenon.

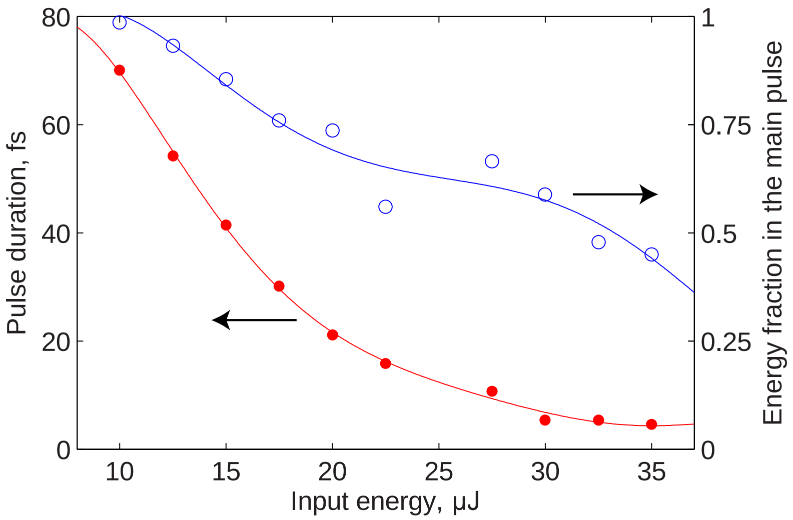

Supplementary Figure 7. Measured pulse duration and pulse quality dependence on the input energy in the case of the shortest pulse configuration. The 20-cm fiber was filled with 3 bar Xenon. Firstly, the pulse duration rapidly decreases with increasing input pulse energy and then saturates at around 32 μJ. However, the energy fraction in the main pulse (integral over the measured temporal profile as compared to a Gaussian pulse of the same FWHM duration and amplitude) slowly decreases in the beginning and starts dropping fast at around 32 μJ, meaning that further increase of the input energy only contributes to the long pedestal without increasing the peak power of the main useful pulse.

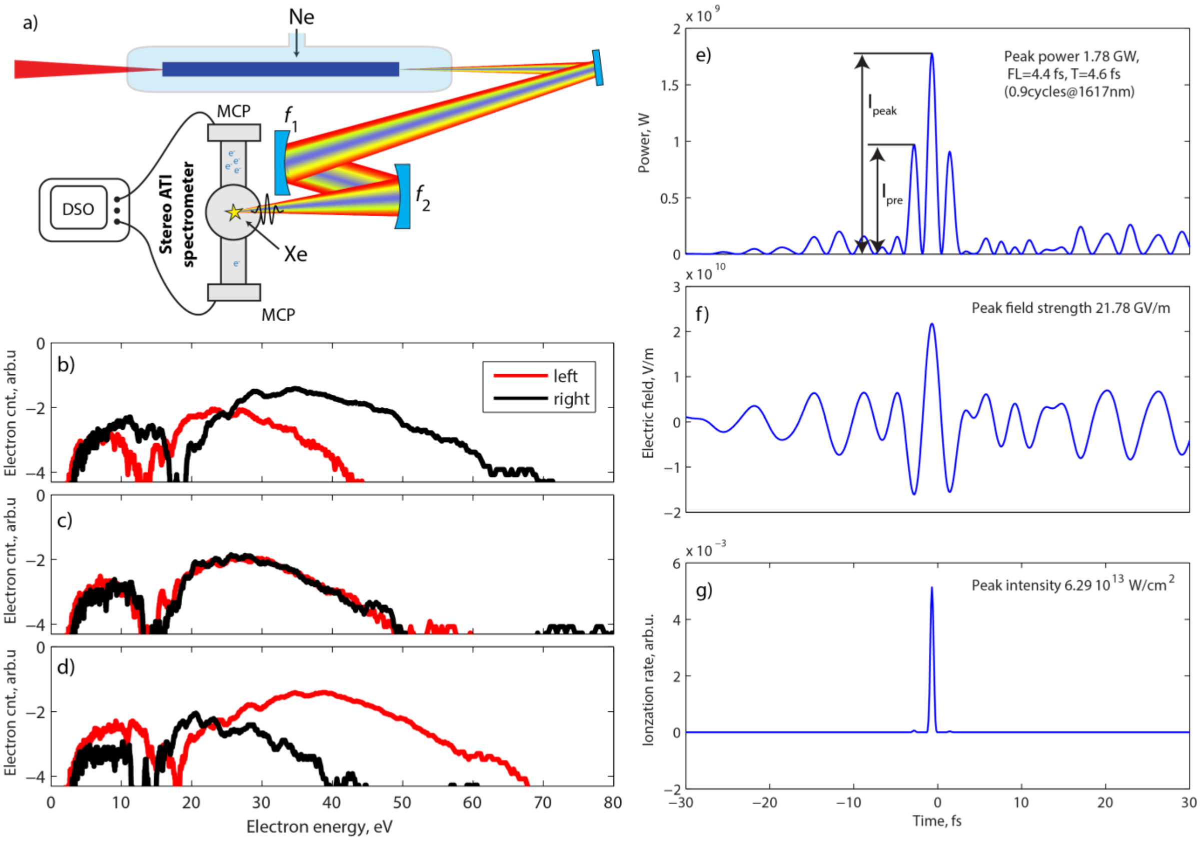

Supplementary Figure 8. An illustration of application of the self-compressed pulses for strong field physics experiments. a) experimental scheme for the use of self-compressed single-cycle pulses for ATI electron spectrometry. DSO – digital sampling oscilloscope. Spherical mirror focal lengths f_1 and f_2 were chosen in order to increase the intensity in the ATI spectrometer. Panels b) and d) show the large asymmetry of the electron spectrum in the two directions for different CEP, indicative of *+cos* and *–cos* electric fields, whereas panel c) shows the symmetric case, indicative of a *sin* electric field. Panel e) shows the instantaneous intensity and the intensity ratio of

the two adjacent half-cycles I_{pre}/I_{peak} and f) electric field of the shortest self-compressed pulse. Panel g) show the corresponding calculated ionization rate in Xe assuming 60 μm spot size.

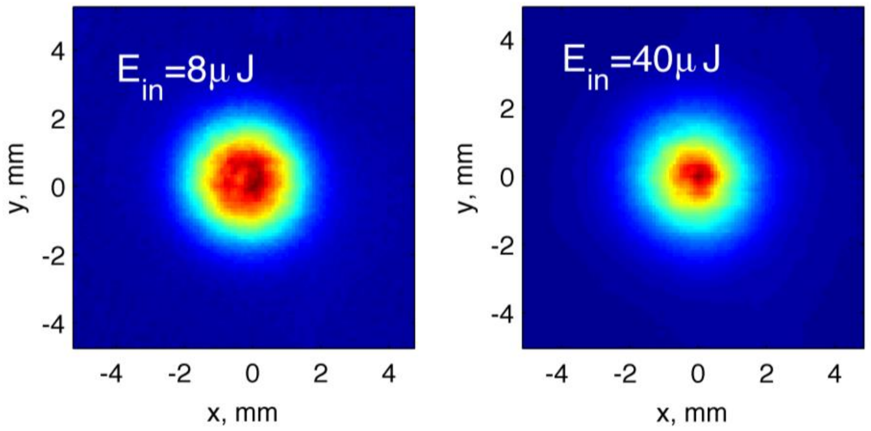

Supplementary Figure 9. Comparison of the beam profile at the output of the fibre for different input pulse energies. Left panel – low input energy case, right panel –high input energy case.

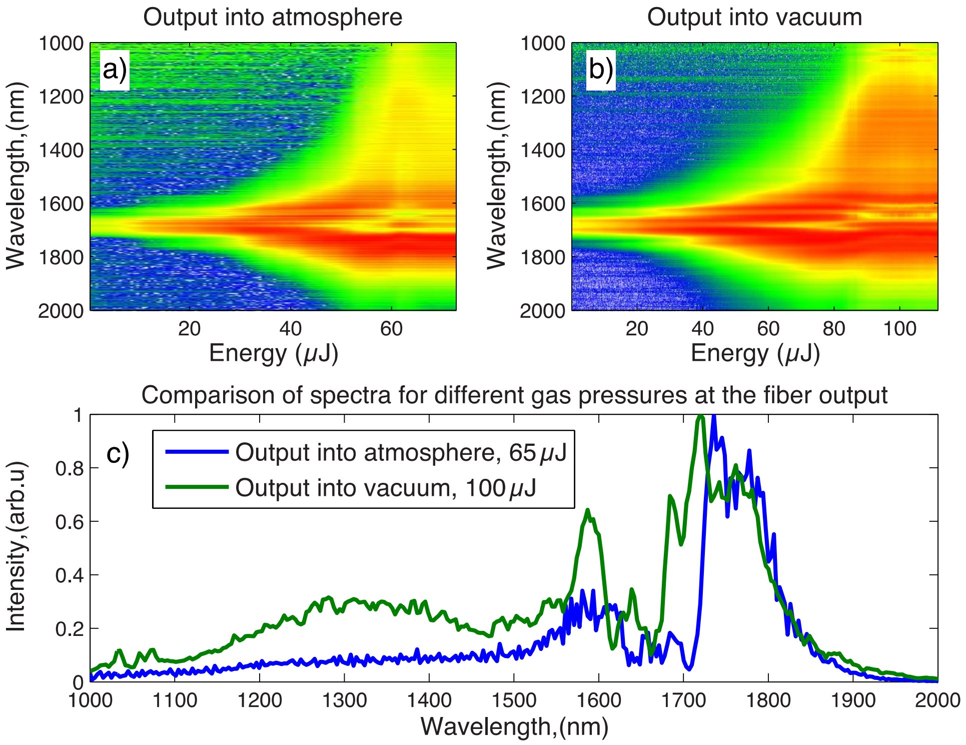

Supplementary Figure 10. Comparison of self-compressed pulse spectrum in case of static cell and pressure gradient. Panels a) and b) show the measured spectral evolution at the output pulse as a function of input pulse energy for a L=45 cm piece of Kagome filled with static pressure of 1.2 bar and with the fiber output end connected to vacuum (pressure gradient case) respectively. c) comparison of broadest spectra in case of small and large pressure gradient in the fiber. Because the highest gas pressure is present only at the fibre input and the pressure at the output is 0, the effective interaction length in the vacuum-terminated case is roughly halved compared to static cell case. Correspondingly, the pulse energy at which the optimum pulse spectral broadening and the pulse compression are expected are significantly increased from 65 to 100 μJ and the spectral broadening is more easily controlled. Consequently, the self-compression scheme based on the Kagome fibre is shown to be capable of serving both typical geometries of a strong field experiment—both with a small dispersion added by transmission components and with a termination directly into the vacuum of a target chamber.

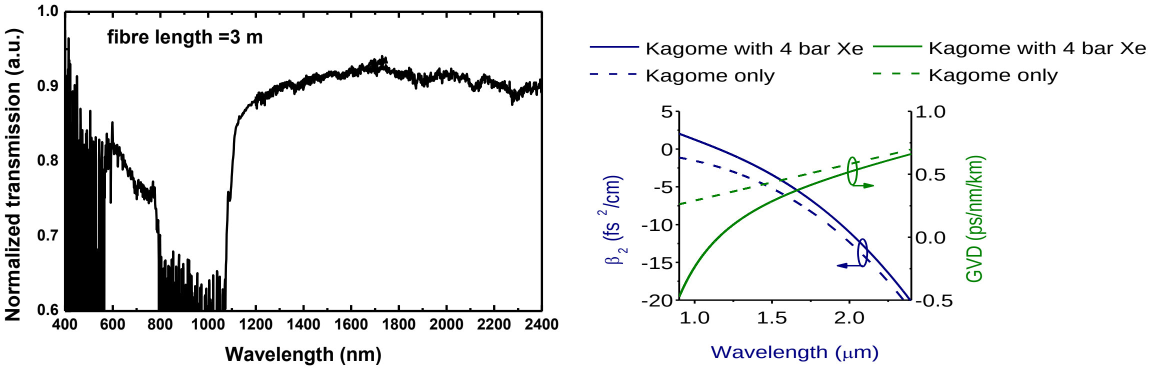

Supplementary Figure 11. Fiber properties. a) 3 m long fibre measured transmission spectrum. The transmission signal is normalised to the whit light source. The spectrum exhibits a very low loss for the wavelengths longer than 1 μm. This corresponds to the fundamental band of the inhibited coupling guiding fibre[1] and extends above 2.4 μm; which is the long-wavelength detection limit of the used spectrum analysers. b) Calculated dispersion profile using finite element method in the spectral range relevant to this experiment.

Supplementary Notes

Waveguide dispersion

The dispersion of the waveguide was modelled using finite element analysis and commercially available software package. The calculated waveguide dispersion and the experimentally measured transmission are summarized in Supplementary Figure 11. The modelled structure was extracted from scanning electron micrograph (SEM) of the fibre. The details of mode solving can found in the supplementary materials of Supplementary Reference[1]. The numerically calculated dispersion results are extremely reliable and are backed with 10 years of very good agreement with group delay measurements.

The polynomial fitting function (3) and its coefficients that describe the dispersion are listed as follows:

$$GDD_{[\frac{ps}{nm\times km}]} = -3335.64 * \Lambda * (0.00931144 - 0.0181275 \times \Lambda + 0.00986497 \times \Lambda^2 - 0.000860359 \times \Lambda^4) \quad (3)$$

Here wavelength Λ is expressed in units of μm. It can be converted to GDD in fs^2/m units using the following conversion factor:

$$GDD_{[fs^2/m]} = GDD_{[\frac{ps}{nm\times km}]} \times \left(\frac{-\lambda^2}{2\pi c}\right) \times 10^{24} \quad (4)$$

Supplementary Discussion

Pulse propagation dynamics

The envelope evolution upon nonlinear pulse propagation in the waveguide is a highly complex process involving an interplay between the nonlinear phase shift due to intensity-dependent refractive index and dispersion of the waveguide. The use of noble gas as a nonlinear medium that has no vibrational or rotational Raman activities provides a nearly perfect instantaneous nonlinearity that works favourably for the formation of optical shock waves.

In the case of non-linear propagation in the self-compression regime, the short pulse is obtained at a specific distance in the fibre for given conditions. However, the fibre length cannot be varied in the experiment. The easily variable parameter is the pulse energy which also determines the position at which the shortest pulse width is achieved. Supplementary Figure 3 shows the measured pulse spectrum and temporal profile dependence on the input energy. Generally, the higher is the input pulse energy, the broader is the spectrum and the shorter is the pulse at the output of the fibre. The experiments agree well with the theory calculations summarized in Supplementary Figure 4.

The dynamics can be roughly divided into three phases. In the first phase, the pulse continously shortens with increasing input pulse energy as indicated in the graph of Fig.3d in the main text. This regime can be considered as a classical high order soliton evolving towards short peak formation. The spectrum broadens nearly symmetrically, the positive chirp due to self phase modulation (SPM) is balanced with the phase shift induced by the anomalous dispersion of the fibre (see Figure 1c in the main text). However, when the pulse gets shorter, the peak of the pulse is slowed down relative to the edges of the pulse due to the dependence of the group velocity on the intensity and an optical shock wave formation sets in enhancing pulse shortening[12]. Finally in the last stage marked (iii) in Supplementary Figure 3 a very short light transient is formed right at the exit of the fibre with the envelope duration shorter than the field cycle with most of the input pulse energy concentrated in it. At this point the peak intensity reaches the level where ionization becomes significant and eventually limits the further increase of peak power.

In order to emphasize the role of shock-waves in our self-compression conditions we simulate pulse propagation with the self-steepening term switched on and off. The representative time-frequency analysis in Wigner representation is shown in Supplementary Figure 5. The self-steepening effect yields a significantly broader pulse spectrum and shorter transient of the self-compressed pulse than it would be in a pure SPM case.

We experimentally found that the pulses at the exit of the fibre are slightly positively chirped which is confirmed by numerical simulations. Since the glass of the window has a negative dispersion in the spectral range of the pulse, the residual chirp of the self-compressed pulses is compensated in the window material. Because the waveguide, like the exit window, also exhibits only anomalous dispersion, we are fortunate to avoid forming the shortest pulse while it still propagates inside the waveguide core. As pointed out in the methods section of the main manuscript, this measure helps raising the energy level that can be safely transmitted through

the waveguide in the self-compression regime. This is possible because although the window contributes a tiny dispersion fraction, it accounts, nevertheless, for a significant shortening of the temporal pulse profile and an approximately 30% increase of the peak power, as shown in Supplementary Figure 6c. Similar effects on the pulse shape of the self-compressed pulses due to window were observed in the case of filamentation[9].

Supplementary Methods

Pulse generation and characterization experimental setup

The experimental setup consists of a femtosecond IR optical parametric amplifier (OPA), Kagome lattice fibre enclosed in a gas cell and an apparatus for spatially encoded spectral phase interferometry for direct electric-field reconstruction (SEA-SPIDER) used for conventional pulse characterization. Supplementary Figure 1 depicts the detailed schematics of the optical arrangement, excluding the ATI spectrometer used in the demonstration of the strong-field capability addressed later. The output of the OPA pumped by a solid-state Yb:CaF_2 chirped pulse laser amplifier running at 2 kHz is tuneable in the 1.4-1.9 μm range and produces transform limited pulses of 80 fs duration across the tuning range. The dispersion estimation of the fibre show that for gas pressures used in the experiment the waveguide stays in the negative dispersion regime at wavelengths λ>1.1 μm. This allows formation of high-order solitons and ultimately leads to the formation of an optical shocks resulting in pulse self-compression at a certain distance. The position where the shortest pulse is formed can be controlled by changing the input pulse energy. More details on the pulse scaling and optimization are given in Supplementary Methods section "Optimization of self-compression and energy scalability".

Pulses from the OPA centered at 1.8 μm wavelength are coupled into the Kagome fibre. The chirp of the input pulses from the OPA is fine-tuned using a prism compressor. The evolution of the pulse temporal profile as a function of input pulse energy was recorded using the SEA-SPIDER technique. The SEA-SPIDER setup was optimized for mid-IR spectral range by using ancillary beams derived directly from the laser pulse at λ_0 = 1.03 μm. To obtain a frequency shear between the two ancillary beams, they were passed through two independently tuned 3-nm narrowband interference filters. As a result, the wavelength of one ancillary beam is 1025 nm whereas the other ancillary beam is at 1035 nm. The two ancillary beams and the self-compressed test beam are focused using a common spherical mirror onto a BBO crystal, resulting in the generation of two non-collinear sum-frequency beams. The two sum-frequency signals are subsequently spatially filtered and re-imaged on the imaging spectrometer[2]. By processing the measured interference pattern the full temporal profile is reconstructed. The setup was carefully calibrated and cross-checks were applied by simultaneously recording FROG traces so long the bandwidth of frequency-broadened pulses remained within the working bandwidth of the FROG apparatus. The SEA SPIDER and FROG pulse reconstructions under such conditions produced a very nice agreement. Despite the use of a noncollinear beam geometry the SEA-SPIDER technique is free of geometric time smearing. Another factor that makes it a technique of choice for measuring over-an-octave bandwidth pulses with potentially a sub-cycle duration is the lack of bandwidth limitation to within a single octave. The sum-frequency signals are generated from the interaction with narrowband ancillary beams, whereby the entire test pulse volume, despite the noncollinear arrangement, is fully

overlapped with the ancillary beams that carry long narrowband pulses and are of a relatively large diameter. The two sum-frequency beams are then filtered using simply a spatial filter and re-imaged on the imaging spectrometer. The full temporal profile of the self-compressed pulse is reconstructed by processing the measured interference pattern of the two ancillary beams on the imaging spectrometer slit.

The SEA-SPIDER technique allows reconstruction of the temporal pulse profile across one transversal spatial coordinate. These measurements, in combination with the direct beam profile measurement using a pyroelectric camera confirm that the pulse self-compresses uniformly across the beam.

As a crosscheck of the SPIDER measurements, we characterized the dynamics of the pulse profile evolution using SHG FROG technique. The results are summarized in the Supplementary Figure 2. Although the experimental conditions are slightly different, these measurements confirm that the pulse uniformly self-compresses at this high peak power regime forming a short pulse with most of the energy concentrated in it. The experimental parameters were not optimized, namely the input pulses were centred at 1.6 μm, there was some uncompensated chirp of the OPA source, which resulted pulses with stronger pedestal. The limitation of FROG technique is that the bandwidth is limited to below one octave. In other words, for the over-an-octave bandwidth, the second harmonic of the long wavelength wing overlaps with the short wavelength spectral wing of the fundamental pulse. In the non-collinear geometry pulse duration is inevitably overestimated due to geometrical smearing effect. Despite these limitations, pulses as short as 8 fs were measured using SHG FROG technique independently of SEA-SPIDER measurement.

Characterization of the self-compressed pulses with stereo-ATI electron spectrometry

We characterized the self-compressed pulses using stereo Above Threshold Ionization (stereo-ATI) electron spectrometry[3] and demonstrate an example of the application of these self-compressed pulses for strong field experiments. Using stereo ATI spectrometer that consists of two time-of-flight (TOF) electron spectrometers in two directions we measure the photoelectron spectrum of the ionized noble gas atoms (Xe) for different carrier envelope phase (CEP) values. The calculated instantaneous intensity (field squared) of the shortest self-compressed pulse and the ionization rate of xenon as shown in Supplementary Figure 8. By focusing that into 60 μm spot, the peak intensity 5 × 10^{13} W/cm^2 is reached. Due to highly non-linear nature of the ionization, only one half-cycle yields significant ionization rate, leading to a cleaning effect of the wings.

The CEP dependent ATI spectrum measurement is a tool that provides direct information on the number of cycles in the ultrashort pulse[4]. For a cosine pulse ($\varphi_{cep} = \pi/2$), there is only one strong half-cycle that generate electrons that are drifting only in one direction. The other half-cycles have much lower instantaneous intensity and produce much lower electron yield. Moreover, in the energy resolved photoelectron spectrum one can directly measure the cut-off which is proportional to the instantaneous intensity $U_p \propto I_{inst} \times \lambda^2$. There are several ways to extract the information about the duration of the envelope of the pulse. Here we estimate the number of cycles in the pulse by measuring the ratio of the cut-offs in the left and right directions U_p^{left}/U_p^{right}, this way directly measuring the ratio of the instantaneous intensity of the adjacent half-cycles[4]. For a sub-cycle pulse the intensity of the adjacent half-cycle is <50%. The shortest pulse generation and the strong-field ATI spectrometry experimental conditions were slightly different. For the ATI experiment the fibre length was optimized for high energy. Therefore the pulses are slightly longer, producing pulses with the adjacent half-cycle peak

intensity ratio of around 60% as shown in the measured electron spectrum in Supplementary Figure 8.

One challenge of the nonlinear pulse self-compression is that the pulse intensity in the last stage of the compression, just before the fibre exit, is at its highest. The high intensity compatible with the level needed for strong-field experiments can be achieved by filling the fibre with high ionization potential (I_p) noble gas and tightly refocusing the self-compressed beam on target. In the experimental scheme shown in Supplementary Figure 8 a), we filled the fibre with Neon gas and the lower I_p Xenon gas was used in the ATI spectrometer. The beam was refocused with demagnification factor $f_1/f_2=2$ thus further increasing the intensity in the interaction region. This allowed nearly fully ionizing the target gas while completely avoiding detrimental effects due to ionization in the fibre.

Numerical pulse propagation modelling

The model used in this work is based on the generalized nonlinear Schrödinger equation (GNSE)[5,6] modified to include ionization effects induced by high-intensity laser fields[7,8]. To study the evolution of ultrashort laser pulses toward sub-cycle field waveforms, we represent the GNSE for the electric field as

$$\frac{\partial}{\partial z}A(\omega,z)=\left[i\hat{D}(\omega)-\alpha(\omega)\right]A(\omega,z)+\hat{F}\left[i\frac{\omega_0}{c}\hat{T}\left(\sum_{i=1}^{5}n_{2i}\left|A(\eta,z)\right|^{2i}\right)A(\eta,z)-\right.$$
$$\left.-\frac{i\omega_0}{2c\rho_c\hat{T}}\rho A(\eta,z)-\frac{(\rho_0-\rho)U_iW}{2I}A(\eta,z)-\frac{\sigma(\omega_0)}{2}\rho A(\eta,z)\right] \quad (1)$$

Here, $A(\eta, z)$ is the complex amplitude of the electric field, $A(\omega, z)$ is its Fourier transform, η is the retarded time, ω is the frequency, z is the propagation coordinate, $I(\eta, z) = |A(\eta, z)|^2$ is the field intensity, $\hat{D}=\beta(\omega)-\beta(\omega_0)-\partial\beta/\partial\omega|_{\omega_0}(\omega-\omega_0)$ is the dispersion operator, ω_0 is the central frequency of the input laser field, $\beta(\omega)$ is the propagation constant, $\alpha(\omega)$ is the linear loss, n_{2i} are the Kerr-effect coefficients, $\hat{F}$ is the Fourier transform operator, c is the speed of light in vacuum, $\hat{T}=1+i\omega_0^{-1}\partial/\partial\eta$ is the shock operator, ρ is the electron density, W is the photoionization rate, $U_i = I_p + U_{osc}$, I_p is the ionization potential, $U_{osc}=e^2I/(2\varepsilon_0 cme\omega^2)$ is the energy of field-induced electron oscillations, $\rho_c=\omega_0^2 m_e\varepsilon_0/e^2$ is the critical plasma density, m_e and e are the electron mass and charge, respectively, ρ_0 is the initial density of neutral species, and $\sigma(\omega)=e^2\tau_c[m_e\varepsilon_0 c(1+\omega^2\tau_c^2)]^{-1}$ is the inverse bremsstrahlung cross section, and τ_c is the electron collision time.

In our model, the field evolution equation (1) is solved jointly with the equation for the electron-density buildup,

$$\frac{\partial\rho}{\partial\eta}=W(\rho_0-\rho)+\frac{\sigma}{U_i}\rho I\ , \quad (2)$$

which includes photoionization and impact ionization[5,6].

The fiber dispersion was calculated using the finite-element method (see Supplementary Notes). The parameters used for modeling are: $n_2 = 5.9\cdot10^{-7}(p/p_{atm})$ cm^2/TW, $n_4 = -2.15\cdot10^{-8}(p/p_{atm})$ cm^4/TW^2, $n_6 = 5.6\cdot10^{-10}(p/p_{atm})$ cm^6/TW^3, $n_8 = -6.8\cdot10^{-12}(p/p_{atm})$ cm^6/TW^3, $n_{10} = 3.2\cdot10^{-14}(p/p_{atm})$ cm^6/TW^3, τ_c = 190 (p_{atm}/p) fs, and I_p =12.13 eV (see, e.g., Supplementary Reference[9]), where p is the gas pressure and p_{atm} is the atmospheric pressure. The fibre mode area is taken equal to S_{eff} = 3650 μm^2. The photoionization rate W was calculated using the Popov-Perelomov-Terent'ev formulation of the Keldysh model[8,10].

Optimization of self-compression and energy scalability

The fibre length, gas type and pressure were optimized to ensure the shortest pulse generation. The duration of the self-compressed pulse can be decreased by increasing the corresponding soliton number which translates into shortening the fibre length. Higher soliton numbers help to achieve shorter pulse widths within a shorter stretch of fibre and make the pulse less sensitive to the nonuniformities of the transmission of the fibre as the dynamics is confined to a shorter length. However, the reduction of the nonlinear phase shift due to shortening of the fibre has to be compensated by increasing the nonlinearity of the medium. The gas type is chosen according to the nonlinearity and pressure limitations of the experimental setup. Different inert gases (Xe, Kr, Ar, Ne, He) have different ionization potentials I_p. The higher the I_p, the higher the intensity can be achieved before detrimental ionization effects sets in, the more energy can be coupled into the fibre. However, the nonlinearity of the gas also decreases strongly with increasing I_p. For the experiment, the Xe gas was chosen which has the highest nonlinearity. Another optimization parameter is the gas pressure in the cell – the nonlinearity grows linearly with the pressure. This means that for example, by increasing the pressure twice, almost the same dynamics can be achieved at just half of the intensity. The ionization probability is a highly nonlinear function of intensity. However, in the experiment in order to outcouple the pulse with minimal distortion a very thin window has to be used which limits the pressure that can be used in the gas cell. In the experiment Xe at 4 bar was used. The maximum pressure that we achieved with 150 μm thick window is 10 bar.

The maximum transmitable pulse energy depends on a number of experimental parameters, such as coupling efficiency, nonlinearity of the filling gas, pulse duration, etc. Although we did not do a systematic damage threshold measurements, we did observe damage of the fibre at around 150 μJ of the input pulse energy. However, recently mJ level pulse transmission through a very similar fibre structure was demonstrated[11]. The typical coupling efficiency in our experiments was 85-90%.

Furthermore, our system provides a lot of freedom in scaling both in terms of pulse energy and spectral properties. These control parameters of our system are the following: a) the fibre transmission and dispersion can be tailored by simply changing the cladding silica web thickness. With this, one can tune the dispersion regime and zero-GVD wavelength; b) the fibre dispersion is so low that introducing a gas provides a further dispersion control. c) energy scaling and photo-ionization handling could be done by simply introducing a higher I_p gas such as helium, and/or operating at low pressure. This could be done without compromising the nonlinear spectral broadening by adjusting the fibre length and its effective area.

Another limitation is the spatial self-focusing inside the fibre due to Kerr lens which limits the maximum peak power. The critical power scales with wavelength as $P_{crit} \propto \lambda^2$, therefore the infrared spectral range around 1.8 μm we use in our experiments acts favourably and allows achieving higher peak power of the final self-compressed pulse. The experimental parameters were optimized in order to achieve the shortest pulse duration keeping the peak power below the self-focusing threshold.. The changes of the beam profile at the output of the fibre at low and high input energy are minor in our experiments, as shown in Supplementary Figure 9. We experimentally show the possibility to upscale the energy of the self-compressed pulse by choosing the type of gas and simultaneously adjusting the gas pressure and the fibre length. The pulse compression in the Kagome fibre can be optimized for various scenarios and the extremely short pulse duration comes at the expense of reduced fidelity of the pulse (see Supplementary Figure 7 for the pulse quality dependence on input pulse energy). The shortest duration of the self-compressed pulse measuring 4.5 fs FWHM (Fig. 3, main text) was obtained

in a 20-cm long piece of fibre filled with Xe (I_p=12.13 eV) at the pressure of 4 bar and the corresponding input pulse energy was 35 μJ. The highest output energy of 75 μJ of the self-compressed pulse in our experiments was obtained in a 45-cm-long piece of fibre filled with Ar (I_p=15.8 eV) at the pressure ~1 bar. These results demonstrate great flexibility of the nonlinear pulse compression scheme whereby the substitution of the gas ionization potential and dispersion can be efficiently compensated by controlling the gas pressure and the fibre length. Nevertheless, there is a noticeable impact on the fidelity of the self-compressed pulse as the shortest duration obtained at the highest energy in the case of Ar measures 6 fs FWHM. The ability to reach an efficient self-compression regime over a wide parameter space including the pulse energy, wavelength and the Kagome waveguide geometry proves the overall robustness of the method and its adaptability to application-dictated target parameters.

Supplementary References

1. Couny F, Benabid F, Roberts PJ, Light PS, Raymer MG. Generation and photonic guidance of multi-octave optical-frequency combs. *Science* **318**, 1118-1121 (2007).
2. Witting T, Frank F, Arrell CA, Okell WA, Marangos JP, Tisch JWG. Characterization of high-intensity sub-4-fs laser pulses using spatially encoded spectral shearing interferometry. *Opt Lett* **36**, 1680-1682 (2011).
3. Sayler AM*, et al.* Real-time pulse length measurement of few-cycle laser pulses using above-threshold ionization. *Opt Express* **19**, 4464-4471 (2011).
4. Haworth CA, Chipperfield LE, Robinson JS, Knight PL, Marangos JP, Tisch JWG. Half-cycle cutoffs in harmonic spectra and robust carrier-envelope phase retrieval. *Nat Phys* **3**, 52-57 (2007).
5. Couairon A, Mysyrowicz A. Femtosecond filamentation in transparent media. *Phys Rep* **441**, 47-189 (2007).
6. Skupin S*, et al.* Self-compression by femtosecond pulse filamentation: Experiments versus numerical simulations. *Phys Rev E* **74**, (2006).
7. Brabec T, Krausz F. Nonlinear optical pulse propagation in the single-cycle regime. *Phys Rev Lett* **78**, 3282-3285 (1997).
8. Keldysh L. Tunneling Theory ofMulti-Photon Absorption. *Zh Eksp Teor Fiz* **47**, 1964 (1945).
9. Berge L, Skupin S, Steinmeyer G. Temporal Self-Restoration of Compressed Optical Filaments. *Phys Rev Lett* **101**, (2008).
10. Yudin GL, Ivanov MY. Nonadiabatic tunnel ionization: Looking inside a laser cycle. *Phys Rev A* **64**, (2001).
11. Debord B*, et al.* Multi-meter fiber-delivery and pulse self-compression of milli-Joule femtosecond laser and fiber-aided laser-micromachining. *Opt Express* **22**, 10735-10746 (2014).
12. Voronin AA, Zheltikov AM. Soliton-number analysis of soliton-effect pulse compression to single-cycle pulse widths. *Phys Rev A* **78**, (2008).
